# Case of Poisoning by Opium, in Which Blood Was Found Effused in the Brain

**Published:** 1826-02

**Authors:** George Jewel

**Affiliations:** Surgeon, &c.


ART. IV.—*Case of Poisoning by Opium, in which Blood was found effused in the Brain.* By GEORGE JEWEL, Esq. Surgeon, &c.

THE following interesting case of a young woman, whose death was occasioned by taking laudanum a short time ago, having been brought before the Westminster Medical Society, and which, in a few particular, although probably not very material points, was (as I have since learnt) inaccurately stated; and, as a garbled account of it has also appeared in the different newspapers, I have deemed it expedient to draw up the following narration of facts as they really occurred, for insertion in the pages of the Medical and Physical Journal. I feel that some apology will be necessary for my apparent prolixity; but, for the reasons above stated, I wish to give all the particulars of the case, as they will also throw some light upon the circumstances which conduced to a state of despondency in the patient; it having been reported that no cause could be assigned for so desperate an act as self-destruction.

Mrs. B—, ætat. twenty-eight years, residing in St. Martin's-lane, consulted me in the month of July, being then in a state of pregnancy, for a complaint which, she candidly said, she suspected was venereal, and which she represented had been communicated to her by her husband. Upon an examination, I found her statement corroborated by the presence of a venereal sore upon the internal surface of the right labium, accompanied by considerable gonorrheal discharge. Notwithstanding the immediate administration of mercury, and the consequent ptyalism which it produced, and the extent to which I conceived, under existing circumstance, it might be carried with impunity, secondary symptoms began to manifest themselves, the disease having doubtless existed some time, by the evidence of a small ulcer which appeared upon the surface of the right tonsil.

After the system had been kept charged with mercury about three weeks, the patient applied, with my concurrence, to Sir

Astley Cooper; who advised that the mercury should now be discontinued, and prescribed for her the compound decoction of sarsaparilla, &c.

Although the disease assumed a favourable tendency, it was evident her spirits gradually became exceedingly depressed; and, notwithstanding my repeated assurances, and those also of my friend Mr. Boyle, (whom I had requested to see her,) of a happy termination of her complaint, her mind became so absorbed with an idea that the disease would never be eradicated, that nothing could remove the impression. This arose, as it has since appeared, from the fearful anticipation that the gentleman by whom she became pregnant, and who then resided in Holland, should return to England, and find her and his child in a state of disease.

The time, however, at length arrived for her accouchment, and I was accordingly sent for. The labour was natural; the infant perfectly healthy; and her recovery was rapid. The dejected state of her mind still continued, with scarcely any variation, and evidently was not influenced by the change of the parturient function.

On the day fortnight after her delivery, late in the evening, I was summoned rather hastily to see her; but, being in attendance some distance off, I did not reach the bedside of the patient until half-past ten o'clock, so that an hour and a half had elapsed after the period of her taking the laudanum. Learning from a second messenger, whom I met before entering the house, that she had been very sick, I procured an emetic of ipecacuanha and tartarised antimony; but, on going into the chamber, I immediately suspected, from the state of insensibility in which she lay, and from a knowledge of the preceding circumstances, what had happened; and my suspicions were soon confirmed by the nurse's producing a wash-hand basin, containing some portion of the contents of the stomach which had been ejected, and in which the opiate effluvia was exceedingly powerful. I immediately administered the emetic I had brought with me; and, at the same time, I sent to Mr. Tweedale, surgeon, of St. Martin's-lane, whose residence was near at hand, requesting his attendance, and that he would bring with him an emetic; which he kindly did. A strong dose of the sulphate of zinc was now given in solution, followed by large potions of mustard infusion; which, together with constant irritation of the fauces with a feather, excited the action of the stomach, and she vomited copiously; after which she recovered her senses sufficiently to acknowledge, on being questioned, that she had swallowed about two ounces of laudanum, procured from different chemists' shops in the neighbourhood. Not being satisfied that we should succeed in dislodging the whole of the poison by emetics, I suggested the employment of the stomach pump; and Mr. Bampfield, having one in his possession, was sent for. This gentleman speedily arrived, accompanied by Mr. Weiss, the surgeon's instrument maker, with a new instrument; and no time was lost in introducing the tube into the stomach. It is necessary I should here observe, that two hours had now elapsed since the opium had been taken. The remaining contents of the stomach were quickly evacuated. Tepid water was then thrown in, and again discharged; and, after two or three effective operations of this kind, there was neither the appearance nor the slightest effluvia indicating the presence of laudanum in the fluid thus evacuated.

Cold affusion was now liberally employed, by pouring water from a jug over the head and neck of the patient; the effect of which, at first, was remarkable. By her own unassisted muscular powers, the poor woman raised herself up, and in a feeble voice requested time to breathe. But this temporary re-action was but of short duration; for she immediately sunk into that comatose state from which she had momentarily recovered.

It was now deemed necessary to throw into the stomach stimulants of ammonia, brandy and water, &c., and injections into the rectum; which last were easily accomplished by the same instrument, according to the directions given with it. The nostrils were stimulated also by a feather dipped in the liq. vol. corn cervi; friction was incessantly employed, and all the means usually adopted upon similar occasions: but, notwithstanding our most indefatigable exertions, the breathing soon became more and more difficult and stertorous; the whole frame shrunk into a state of collapse; and, after one or two convulsive efforts, she expired at five o'clock in the morning, eight hours after swallowing the fatal draught.

*Appearances on an examination of the body, thirty-three hours post mortem.*

Upon cutting through and everting the abdominal parietes, the aspect of the viscera was perfectly healthy; and, on a minute investigation, no morbid appearance whatever, not even the slightest inflammatory blush, could be discovered, either in the stomach or in any portion of the alimentary canal. The thorax being laid open, the lungs were quite sound, and of the usual colour. The heart was pale, bloodless, and flaccid. On removing the calvarium, the vessels of the dura mater looked turgid; those of the arachnoid membrane and pia mater still more so; and, upon an examination of the cerebrum, the whole of the vascular system of the brain appeared enormously tumid, every vessel seemed gorged with blood. On tracing the veins which lay rather more obscure, a breach in two or three distinct points had evidently taken place, and which was clearly indicated by pieces of coagula; one of which, lying at the superior margin of the right lateral ventricle, must have measured upwards of an inch in extent.

Upon the whole, the appearances which presented themselves in this case seemed to bear a very strict analogy to those which are commonly observable on an examination of the cerebral structure, after death by apoplexy.

It is but justice to the ingenious inventor of the stomach pump to add, that the instrument used in the above melancholy case answered every purpose for which it was intended: at the same time, I should be guilty of a direlection of professional duty, did I not suggest that every practitioner should become familiar with its use; for, although its construction is by no means intricate, yet, if a person attempts to employ it without being previously instructed in its mechanism and mode of action, he will not only find himself extremely embarrassed, but, in all probability, will fail in effecting that good which otherwise might result from his humane exertions.

30, *Gerrard-street; Dec. 9th*, 1825.

